# Enamel apatite crystallinity significantly contributes to mammalian dental adaptations

**DOI:** 10.1038/s41598-018-23826-0

**Published:** 2018-04-03

**Authors:** Anna Kallistová, Roman Skála, Miroslav Šlouf, Petr Čejchan, Irena Matulková, Ivan Horáček

**Affiliations:** 1 Institute of Geochemistry, Mineralogy and Mineral Resources, Faculty of Science, Charles University, Albertov 6, Prague, 2 Czech Republic; 2 Institute of Geology of the CAS, v.v.i., Rozvojová 269, Prague, 6 Czech Republic; 3 Institute of Macromolecular Chemistry of the CAS v.v.i., Heyrovského náměstí 2, Prague, 6 Czech Republic; 4 Department of Inorganic Chemistry, Faculty of Science, Charles University, Hlavova 2030/8, Prague, 2 Czech Republic; 5 Department of Zoology, Faculty of Science, Charles University, Viničná 7, Prague, 2 Czech Republic

## Abstract

The monophyodont molar teeth, prismatic enamel and the complexity of enamel microarchitecture are regarded as essential dental apomorphies of mammals. As prominent background factors of feeding efficiency and individual longevity these characters are crucial components of mammalian adaptive dynamics. Little is known, however, to which degree these adaptations are influenced by the crystallographic properties of elementary hydroxyapatite crystallites, the only inorganic component of enamel. In a miniature pig where individual molars differ significantly in duration of their development and in enamel resistance to attrition stress, we found highly significant differences between the molars in the size of crystallites, amount of microstrain, crystallinity and in enamel stiffness and elasticity, all clearly scaled with the duration of tooth calcification. The same pattern was found also in red deer bearing different molar type. The results suggest that the prolongation of tooth development is associated with an increase of crystallinity, i.e. the atomic order of enamel hydroxyapatite, an obvious component of micromechanical property of mature enamel. This relation could contribute to prolongation of dental development, characteristic of mammals in general. The aspects of enamel crystallinity, omitted in previous studies on mammalian and vertebrate dental evolution, are to be taken in account in these topics.

## Introduction

Enamel, the hardest tissue of vertebrate bodies and proper agent of teeth function, is exclusively a mineral matter: it is composed of compact aggregates of hydroxyapatite (HAp) crystallites while the non-mineral components, proteins and water, form less than 5 wt.% only^1^. The process of enamel mineralization is expected to proceed without any essential biogenic intervention^2^ by interactions of amorphous calcium phosphate and a limited number of enamel matrix proteins (EMPs), which form about two thirds of the forming enamel volume. Their interactions and probably also self-assembly processes resulting in supramolecular aggregates (documented by plenty of *in vitro* experiments^3^) are believed to be the sole mechanism organizing the proper enamel mineralization^4–6^.

The crystalographic properties of enamel apatite and microarchitecture of enamel coat are the essential factors of functional quality of teeth and, in consequence, feeding efficiency and life-expectancy of the individual. This is particularly valid for mammals whose diphyodont dentition provides no chance for reparative dental rearrangements during adult age. This disadvantage is compensated by radical innovations by which mammalian dentitions attained outstanding adaptive qualities essentially contributing to evolutionary prospect of that clade. Two of them (both related to a prolonged developmental period charaterizing mammalian constitution) are to be particularly emphasized: prismatic enamel and the monophyodont multicuspidate distal teeth called molars^7–9^. The enamel prisms, spatially condensated strings of crystallites, provide structural scaffolding for a thick enamel coat and for complex microarchitectural arrangements capable of compensating for extreme stress upon a tooth surface. Molars, as a rule the largest teeth of mammalian dentition and the proper agent of a taxon-specific dental specialization, are characterized by a particularly prolonged developmental period: they are initiated simultaneously with teeth of deciduous dentition, yet appear at eruption at adult age only, after deciduous dentition has been completely worn. With functional specialization of the deciduous dentition (such as molarization of deciduous premolars), the developmental time of the distal teeth can be further prolonged, enabling them to grow larger to respond functional demands of even very large adult body size. In general, such a kind of developmental heterochrony is strongly selected in the clades whose feeding depends upon processing of large amounts of food in molariform dentition, namely in herbivores^10,11^. The question is whether the heterochrony of dental development of posterior molars is marked only by differences in their size and morphologic complexity^10,12^ or whether and to which degree it is accompanied also by shifts in aspects of enamel microstructure. There is no doubt that the form and spatial organization of prisms contribute to functional quality of a tooth in an essential way and that the herbivore clades exhibit a plethora of examples of top complexity in this respect^13^. Little is known, however, how this may affect the properties of the elementary crystallites composing the HAp crystals, from which the components of the enamel coat are formed. Elsewhere^14^, we demonstrated that the crystallographic qualities of elementary crystallites significantly change during enamel maturation and are closely related to changes in enamel mechanical properties. Crystallite size, microstrain and microhardness were found particularly significant among the variables that show essential variation in this respect. Crystallite size can be measured as the effective size of the coherently diffracting domains within a polycrystalline material, while microstrain specifies the degree of lattice defects (such as grain boundaries, dislocations, stacking faults, etc.) present in the crystallite^15^. The crystallinity – a complex variable indexing the atomic order of crystallite structure^16,17^ – was only exceptionally measured or even taken into account in context of enamel microstructure^18–20^.

Here, we compared the state of these variables within the particular teeth of a molar row (M_1_, M_2_, M_3_) using a series of laboratory pigs aged 16 to 108 months, and tested the effects of individual age, tooth wear and developmental periods of particular molars. Our results suggest that apatite crystallinity increases with length of enamel maturation and influences the final mechanical quality of the enamel coat in an essential way.

## Results

**Figure 1:**
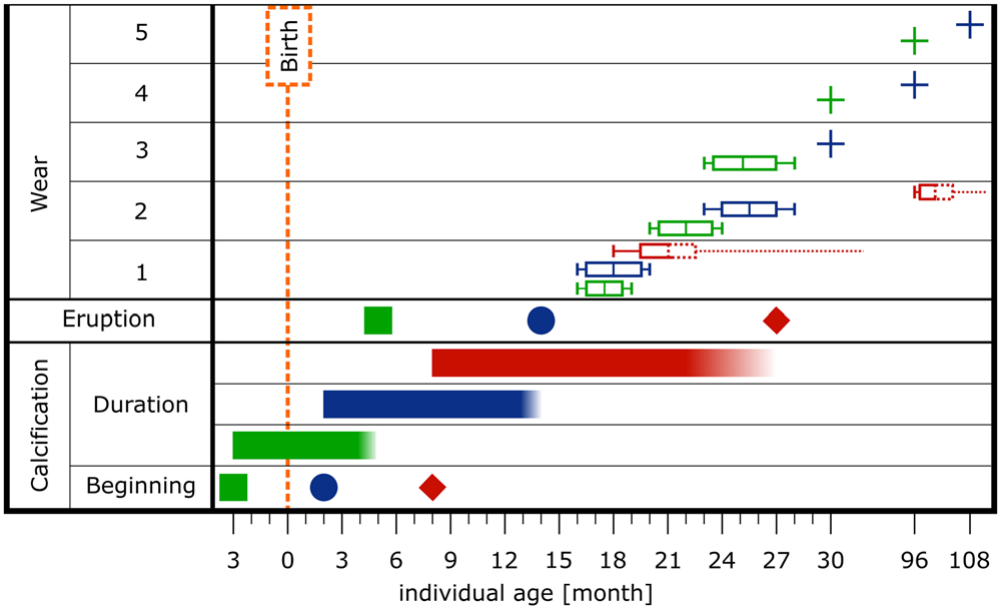
A synoptic survey of developmental dynamics of miniature pig molar teeth (M_1_ - green, M_2_ - blue, M_3_ - red) - individual age of beginning and duration of calcification, eruption and particular stages of tooth wear in terms of categories by Grant:^25^ 1 - no visible cusp wear (category a,b); 2 - minute wear (category b,c); 3 - pronounced cusp wear, visible small dentine fields (category c-e); 4 - advanced cusp wear, extensive dentine fields (stages e-g); 5 - masive tooth wear, most of the crown enamel is missing (category h-m). The boxplot in wear section covers all individuals in particular stage of tooth wear: the youngest individual is in the most left part of the boxplot; the oldest one is in the most right part of the boxplot and the average age of all individuals in particular wear stage is the middle line inside the rectangle. The plus sign was used when only a single individual of particular stage was analyzed.

Our observations show that the M_1_ and M_2_ attained pronounced cusp wear (stage c) around 17 to 18 months after tooth eruption and the advanced wear stage (d-e), with extensive visible dentine fields around 24–25 months for M_1_ and 80 months for M_2_ respectively. On the contrary, 80 months after tooth eruption the M_3_ has only indistinct cusp wear (b) – Fig. 1.

The chemical composition of enamel analysed in five specimens selected across the age spectrum showed a constant pattern varying within limits of experimental error (with small between-individual variations) without any significant correlation with other studied variables. We found relatively broad variations in state of *a* and *c* lattice parameters within the total set of all samples (*a*: 9.4484–9.4526 Å; *c*: 6.8735–6.8879 Å) yet without significant effects from any contextual variable and with no significant dissimilarities or correlations among groups of individual molars (M_1_ vs. M_2_ vs. M_3_) and/or groups of old and young individuals.

**Figure 2:**
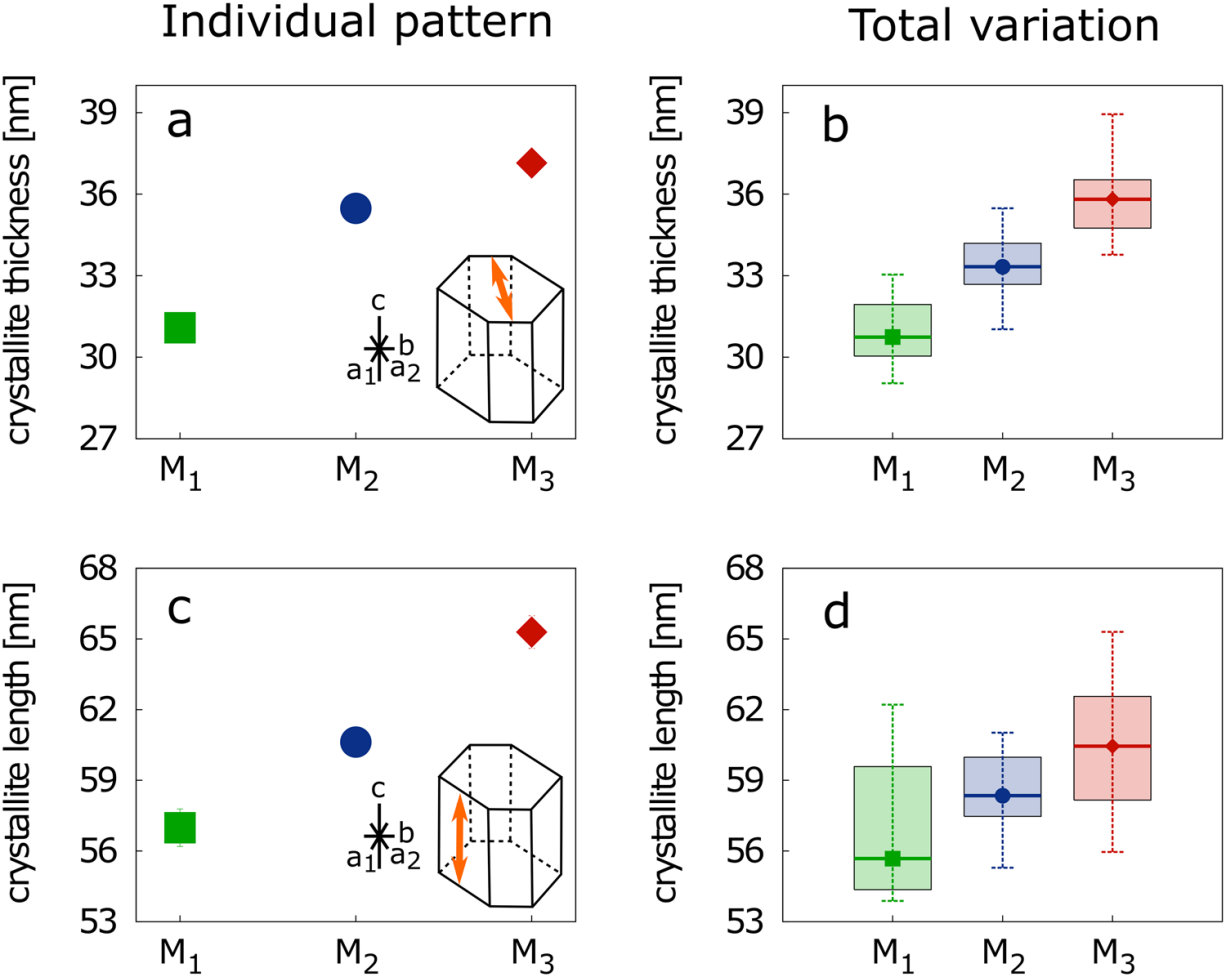
Computed crystallite thickness (**a,b**) and length (**c,d**) of: (**a,c**) 25-month old individual and (**b,d**) all studied samples. Diagrammatic representation (inset) of a hydroxyapatite crystallite with orange arrow showing its (a) thickness and (c) length. Note: The error bars at (a,c) are smaller than the size of symbols.

**Figure 3:**
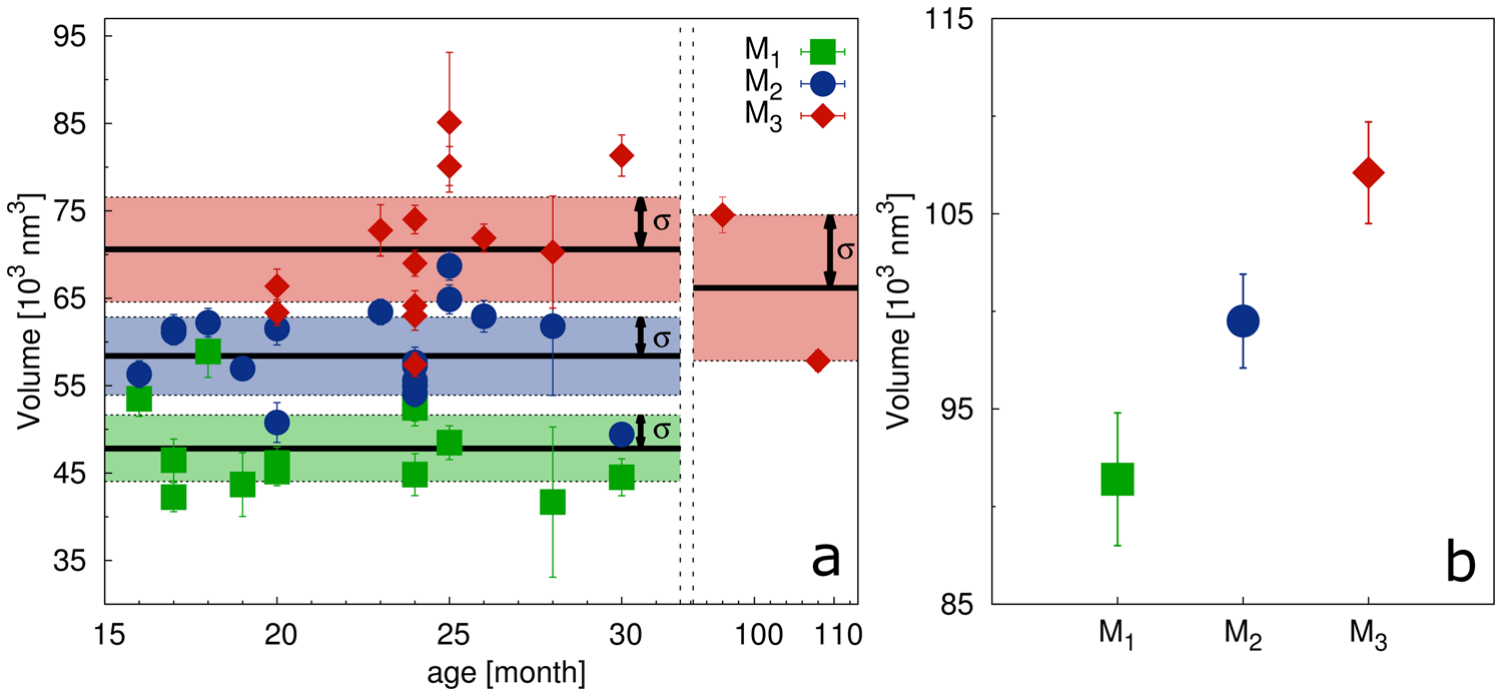
Computed volumes of enamel HAp crystallites of (**a**) miniature pigs. For comparison of changes in crystallite volume with age, the volumes are plotted against the age of each individual. The solid black lines are the average volume for each molar type and the colored areas show a dispersion of data set (1 *σ*). Each point in the plot (**b**) represents the average HAp crystallite volume of individual molar types of red deer.

Crystallite sizes were computed anisotropically since their shape appeared elongated. Thickness ranged between the 290–330 (M_1_), 310–355 (M_2_), and 338–390 Å (M_3_) and length varied between the 538–622 (M_1_), 553–620 (M_2_), and 560–691 Å (M_3_) (Fig. 2). The crystallite volume progressively increased in sequence from M_1_ via M_2_ to M_3_ of each individual with high significance (Supplementary Table S2a). The groups of individual molars (M_1_,M_2_,M_3_) formed distinct entities in regards to crystallite volumes within the range of an age of studied pigs (Fig. 3a). Figure 3b demonstrates the same trend observed in molars of red deer. The third molars of individuals younger than 20 months were still in the embryonic stage of development (i.e., did not reach full adult size) and thus were excluded from our study. The results of the statistical analysis are summarized in Supplementary Tables S1 and S2.

**Figure 4:**
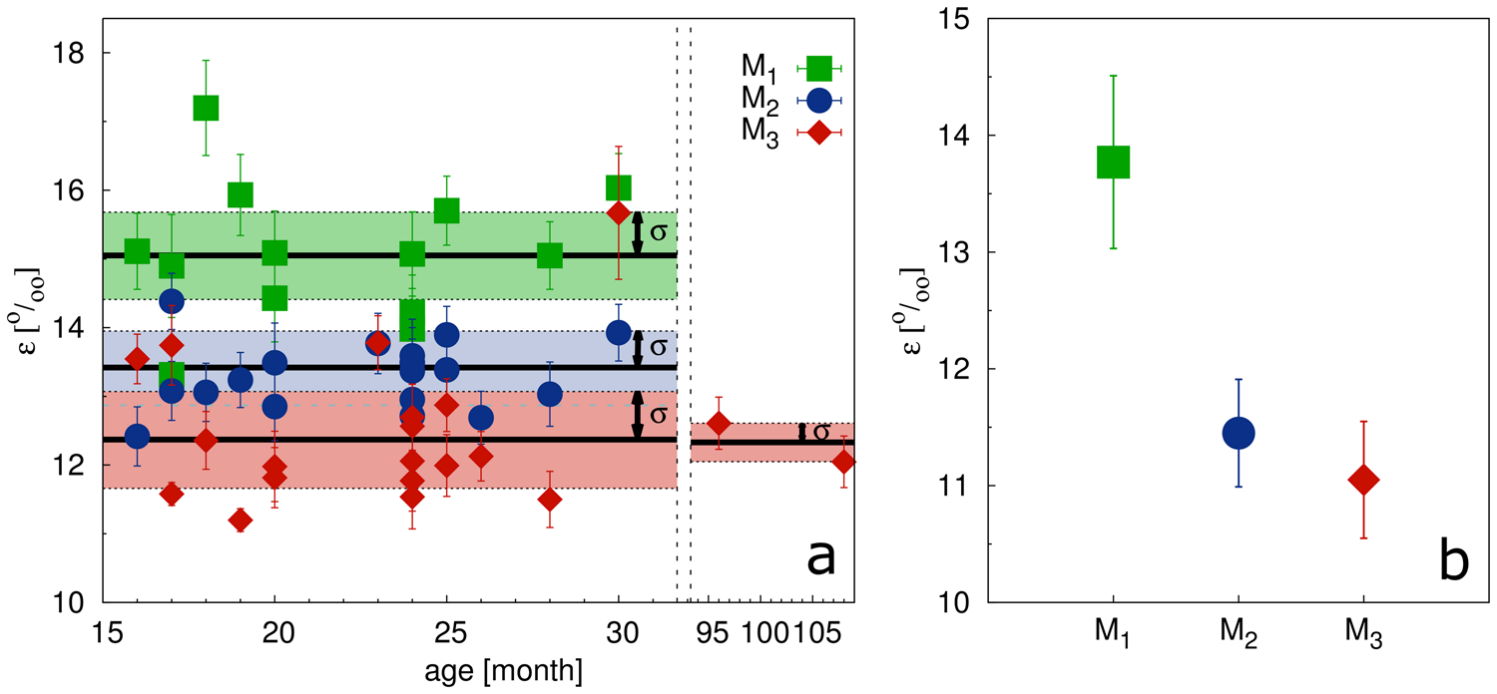
Values of the microstrain (*ε*) of (**a**) miniature pigs and (**b**) red deer.

In contrast to elongated crystallites, the microstrain was isotropic. The absolute values of microstrain ranged between 11.2 and 17.2. The microstrain gradually decreased between individual molars from M_1_ to M_3_ (Fig. 4a). The significance of that trend was confirmed by a one-sided paired t-test, a one-sided two-sample paired Wilcoxon test and a Kolmogorov-Smirnov test. P-values are summarized in Supplementary Table S2b. The red deer samples also followed the decreasing trend of microstrain (Fig. 4b).

**Figure 5:**
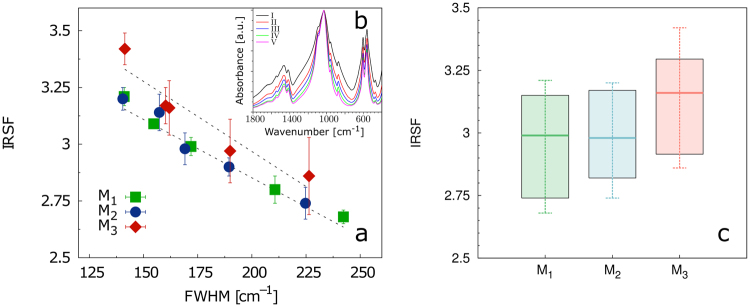
The FTIR spectral differences of phosphate vibrations in the enamel apatite of the five fractions (differ in particle size) of three molar types (M_1_ to M_3_) in the series of tested pigs. (**a**) grinding curves the relation of IRSF against FWHM for three different molar types (each point represents the averaged value of five studied pigs.); (**b**) an example of the measured FTIR spectra used for the plotted parameters (FTIR spectra of five different fractions of second molar (M_2_) of 26 months old individual showing the variations in FWHM); c) IRSF boxplots of the finest fraction values obtained from all studied individuals.

The structural information on HAp was further supplemented with examination of atomic disorder using infrared spectroscopy. Figure 5a shows the particle size dependency of the width of infrared spectra (FWHM) and infrared splitting factor (IRSF). The plot documents higher values of IRSF for M_3_ for all examined sample fractions. The finest fraction (V.) was used to evaluate the atomic disorder. Figure 5c, M_3_ clearly displays the lowest degree of atomic disorder (i.e., the highest crystallinity) among all molar types.

**Figure 6:**
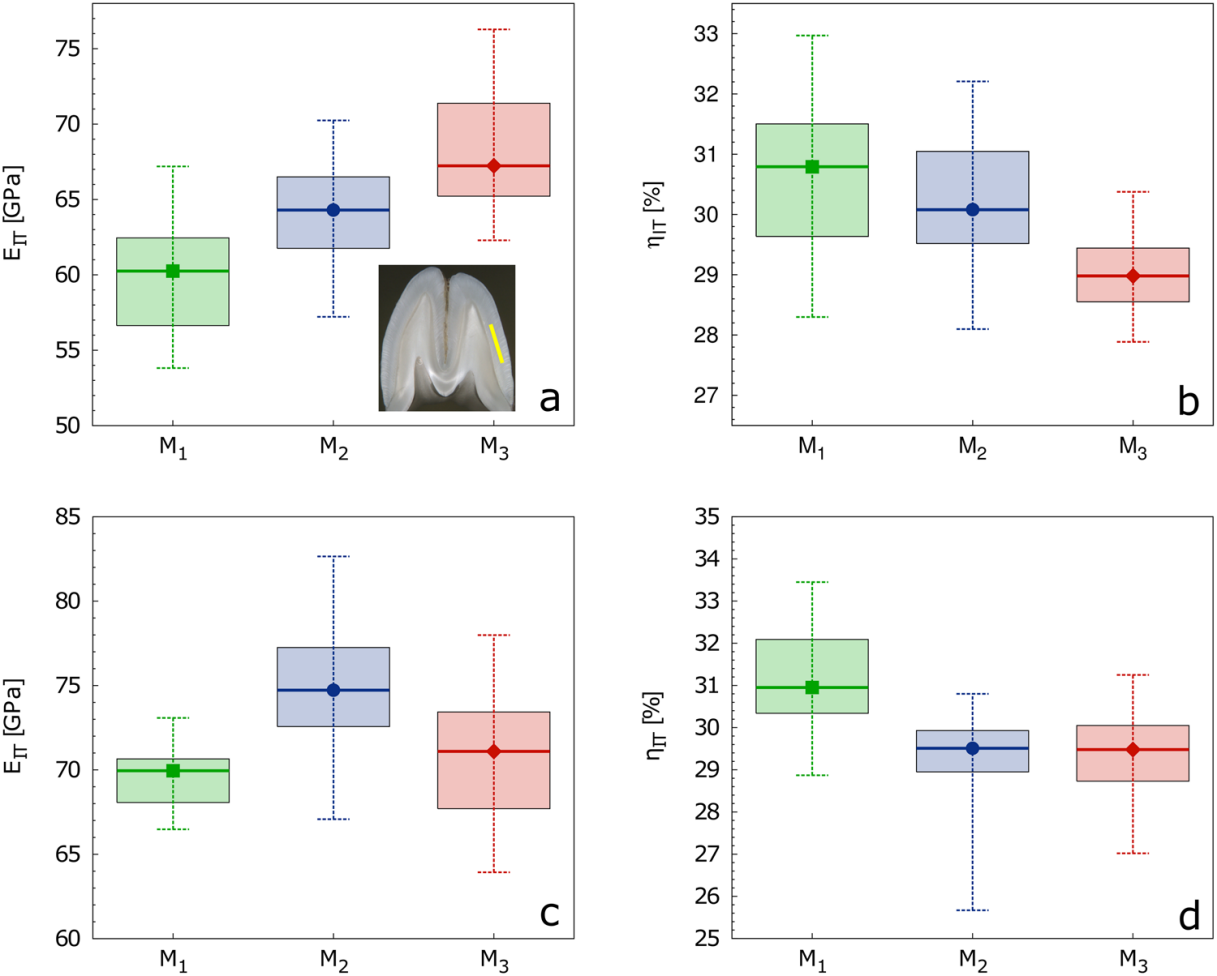
Boxplot (median, min-max, quartiles I-III) of selected micromechanical parameters: (**a**,**c**) *E*_IT_; (b,d) *η*_IT_ of (**a,b**) miniature pigs and (**c,d**) red deer.

The four variables representing the overall mechanical quality of enamel coating: *H*_IT_, *η*_IT_, *C*_IT_ and *E*_IT_ (for their definition, see section Methods) showed different relations to the contextual variables. While the *H*_IT_ and *C*_IT_ remain invariant, the pronounced gradual increase in *E*_IT_ is accompanied by a decrease in *η*_IT_ values especially in the case of M_3_ (Fig. 6). Results of the statistical evaluation are summarized in Supplementary Table S2c,d. It is obvious that the *E*_IT_ values differed significantly between M_1_, M_2_ and M_3_. The comparison of the *η*_IT_ values for M_1_/M_2_ against the M_3_ was also highly significant, but the difference between M_1_ and M_2_ was not sufficiently conclusive.

We used only M_3_ to evaluate both microstructure and microhardness and their dependence on the age of an individual (i.e., on the mechanical degradation/abrasion of HAp) because the M_1_ and M_2_ of old individuals lack enamel coating. Crystallite volume and microstrain of young (i.e., 16–30 months) and old (i.e., 96–108 months) pigs did not show significant differences (Figs 3a, 4a). The statistical analysis based on the alternative hypothesis that the crystallite volume and microstrain of third molars are age independent (for p-values see Supplementary Table S2e), confirmed these results. Likewise, no correlation between the age of individuals and the lattice parameters or chemical composition of all measured HAp was found. On the contrary, the *H*_IT_ and *E*_IT_ values were positively correlated with age, with high significance (Supplementary Table S2f,g).

## Discussion

Individual molars (M_1_, M_2_, M_3_) of pig dentition represent distinct entities either in terms of their morphology or functional qualities and their developmental dynamics. They differ in duration of their developmental period^21,22^, timing of eruption^23,24^, and last but not least in resistency to mechanical stress and tooth abrasion. The most distal molar (M_3_) with the period of calcification more than twice longer that M_1_ show essentially higher resistance to tooth wear: cf. stage b of tooth wear (in sense of Grant^25^) that appears on average at 69 months after eruption in M_3_ against 16 months on average in M_1_. Together with a large tooth size and thick enamel, which enable sufficient enamel sampling for X-ray analyses^14,26^, all these regards make the laboratory pig nearly ideal model for confrontations of the crystallographic variables with various aspects of a tooth function and developmental dynamics. The difference between individual molars in crystallite volume, microstrain, level of crystallinity and aspects of microhardness, revealed in this study, can thus be discussed in regard to the above mentioned contextual variables. Out of them, the individual age, tooth size and degree of tooth wear showed only insignificant effects upon the state of structural variables under the study. In contrast, the differences between individual molars in duration of their calcification corresponded to their differences in state of particular crystallographic variables quite robustly.

A strong positive correlation between the duration of the calcification period and the crystallite sizes/volumes and the negative correlation with the microstrain suggest possible causal relations among these variables, i.e., the longer the enamel calcification, the larger volume and the less lattice imperfections of elementary crystallites. The results of infrared spectroscopy, indexing a degree of crystallinity, revealed the same picture. It is important that two components of microhardness exhibit clearly the same relations as well: the stiffness, which gradually increased with the length of calcification period and the elastic capacity of enamel, which decreased. A comparison of the mesial and distal molars (M_1_ vs. M_3_) is particularly impressive. The enamel of the first molar, the integration of which to deciduous dentition forces earlier termination of its developmental period, is formed by the smallest crystallites irrespective to the amount of lattice defects and atomic disorders, which negatively influences its mechanical property and leads to the severe abrasion already at a young age of the animal. In contrast, delayed eruption of the third molar enables considerable enlargement of tooth size with a thick enamel composed of the largest crystallites with essentially reduced amount of lattice imperfections and atomic disorders. This is positively reflected in the stiffness of the final enamel and its highest resistance to mechanical damage. Worth mentioning is that the same patterns were found also in the molar teeth of red deer which differ from bunodont teeth of pig both in their morphological setting (selenodont molar form) and derived type of their enamel microarchitecture^10,11,27,28^. The common pattern suggests, hence, that the above mentioned relations between the duration of calcification and aspects of crystallinity are invariant to differences in a form of teeth and its enamel microarchitecture.

The relationship between crystallinity and mechanical properties in general are explained by the Hall-Petch relationship (H-PR)^29,30^. In agreement with empirical evidence, it predicts that decreasing crystallite size increases the amount of grain boundaries acting as locking points impeding spatial propagation of dislocation and thus increases the dislocation density within a crystallite. The material with smaller crystallites needs to apply higher stress to move the dislocation across a grain boundary and such a material then has higher yield strength hence Young modulus. For nanocrystalline materials with crystallite size below a critical value (20 nm), the H-PR ceases to be valid^31^. The yield strength and Young modulus can be related to *H*_IT_ and *E*_IT_ measured by microindentation techniques as defined by Ostafinska *et al*.^32^, and the dislocation density can be related to the microstrain as defined by Williamson & Smallman^33^. We should also mention the general behavior of polymer materials outlined by Šlouf *et al*.^34^, which exhibit the positive correlation of crystallinity with hardness, stiffness and elastic part of the indentation work.

Yet, in contrast to microstrain, the yield strength *H*_IT_ of pig enamel shows no significant differences between all three molars differing significantly in their crystallite sizes. It can be quite minute only. Moreover, the *E*_IT_ follows the opposite trend than expected from H-PR. Two explanations are possible: (1) the crystallite size of enamel reached the critical value mentioned above, (2) the dislocation density (microstrain) is too low to reach the lockdown effect and the amount of lattice imperfections together with decreasing crystallite size on the contrary contribute to the deterioration of enamel stiffness. Especially the latter is supported by infrared spectra showing higher crystallinity (atomic order) for molars with a lower amount of lattice imperfections and with higher stiffness of enamel, which is in accordance with Šlouf *et al*.^34^.

It should be noted that also other factors, such as the inner architecture of mature enamel or the content of organic residues can atribute to the mechanical behaviour of enamel coat^35–39^. In regard to our study the former variable can be considered as an invariant - no posteruptional changes in enamel architecture can be expected^14^. Moreover, the observations of Cuy *et al*.^40^, Braly *et al*.^41^ and Fonseca *et al*.^42^ suggest that the hardness and Young’s modulus of enamel apatite are only weakly dependent on the enamel architecture. In contrast, the organic component of the mature enamel (concentrated particularly along EDJ^43^) seems to undergo the age-related changes, e.g. aspartic acid racemization^44,45^. The scarce data available on that topic suggest that the proteins of mature enamel are capable to affect mechanical quality of enamel coat by dissipating a considerable amount of deformation forces by gradual unfolding of their domain structures^36,37^ and reduce deeper propagation of cracks^39^. Hence, the amount of protein may significantly contribute to mechanical qualities of enamel (at least in synergy with other factors) and exhibit certain age-dependent variation as indicated also by our results (see section Results, last paragraph).

Nevertheless, the content of protein in the mature enamel takes about 1% of enamel weight only (0.5–3%)^43,46^ and for obvious reasons it hardly can play a role of prevailing factor of enamel hardness. It would be beyond scope of this paper to hypothesize further on patterns of synergy and roles of particular factors causing the extraordinary mechanic qualities of mammalian mature enamel. We focused just on one of them and convincingly demonstrated that the basic crystallographic properties of enamel crystallites can significantly contribute to the mechanical quality of enamel and are scaled by duration of enamel calcification. For comparative and functional analyses of mammalian dentitions, traditionally operating with teeth shapes, prism arrangements and schmelzmusters^13,27,35^, it provides a new variable worth serious analysis.

## Conclusion

We tested the relationship between duration of enamel calcification and the overall quality of adult enamel coating in laboratory miniature pigs. Our results suggest that from M_1_, through M_2_ to M_3_, the crystallite size, crystallinity and stiffness increase, while the microstrain and elastic part of the indentation work decrease. All these findings lead to the conclusion that the prolonged calcification provides the bigger crystallites with less lattice defects and a higher atomic order, resulting in better mechanical properties of enamel and therefore the increased resistence to tooth wear. In these terms, enamel crystallinity can significantly contribute to the functional qualities of a tooth. It might present a factor contributing to selection for prolonging tooth development, a quality quite pronounced in many mammalian clades and, compared to other vertebrates, characterizing mammals in general.

## Methods

The 20 laboratory miniature pigs (females aged between 16–108 months) originated from the same breed were provided by the Institute of Animal Physiology and Genetics in Lib $\breve{{\rm{e}}}$chov. We were allowed to extract jaws from the animals sacrificed on diverse terms in 2013 and 2014 for purpose of several research projects conducted by the Institute (cf. e.g. Vodička *et al*.^47^, Baxa *et al*.^48^, and Planska *et al*.^49^ etc.). All components of the respective projects, including all procedures, were carried out in accordance with the Projects of Experiment approved by the Animal Care and Use Committee of the IAPG CAS, v.v.i. (Liběchov, Czech Republic), following the rules of the European Convention for the Care and Use of Laboratory Animals and related Czech regulations (see Kallistová *et al*.^14^ for details).

Regarding to a platform of developmental data established in our previous paper^14^ also here our comparisons are based on mandibular molars (M_1_, M_2_, and M_3_). Their development starts during the fetal stage of animal life, yet individual molars differ considerably in onset of calcification stage, total duration of their embryonic development^21,22^ – see Fig. 1 and timing of their eruption. The first molar appears above gingiva at age of 5–6 months, the second molar erupts at 12–14 months, the third molar around 22–27 months^23,24,50^. The maxillar and mandibular teeth erupt at slightly different times but there is no significant difference between left and right teeth respectivelly^50^. The duration of calcification stages considered here as major contextual variable (Fig. 1) takes on average 8 months in M_1_, 14 months in M_2_ and 19 months in M_3_. In addition, we also examined the molar teeth of red deer (*Cervus elaphus*), another species with a well pronounced time delay between eruption of individual molars: we used mandibles of three adult females deposited in collection of Depertment of Zoology, Charles University Prague. The degree of tooth abrasion was classified using categories proposed by Grant^25^.

The teeth were extracted from mandibles, the central part of all teeth was excised perpendicularly to the bucal-lingual tooth side, thin-sectioned, and embedded in epoxy resin. The remaining enamel crown parts were then manually disintegrated (see Kallistová *et al*.^26^) and fragments of pure enamel were grounded under acetone in an alumina mortar. In a few cases, the first and/or second molars were excluded from this study because of terminal stage of the toothwear with absence of enamel coating or presence of large amounts of tartar.

The X-ray powder diffraction measurements were used to characterize the crystallite size and microstrain. They were performed using a Bruker D8 Discover diffractometer equipped with a linear LynxEye detector and a germanium primary monochromator providing CuK _*α*1_ radiation (*λ* = 1.54056 Å). Data were collected in the 2*θ* range of 5–122 with a step size of 0.013 and a counting time of seven seconds at each step. Le Bail whole-pattern fitting was accomplished using the FullProf program^51^. As the crystallite is considered to be of elongated ribbon shape^52–54^ its size was computed for each individual reflection in order to take into account anisotropic broadening (for details see Kallistova *et al*.^26^). In the present paper, the term length is denoted to the size of crystallite in [00.1] direction (i.e. parallel to the c-axis) and the term thickness represent the shortest dimension of crystallite cross-section (i.e. perpendicular to the c-axis). Compared to alternative computation methods^55–58^ the present one exhibited far the best statistic resolution. Unfortunately it did not enable to easily discriminate between the anisotropy related to a-axes (thickness) and that of b-axis (width). Consequently, the comparative analyses are preferably based on the variable of crystallite volume which covers sizes computed in all crystallographic directions.

The program generates the shape of the crystallite in terms of spherical coordinates. To calculate the volume of the crystallites the data were converted into Cartesian coordinates applying the following equations:

1 $$\begin{array}{l}{x}_{i}={r}_{i}\,\sin \,{\theta }_{i}\,\cos \,{{\phi }}_{i}\\ {y}_{i}={r}_{i}\,\sin \,{\theta }_{i}\,\sin \,{{\phi }}_{i}\\ {z}_{i}={r}_{i}\,\cos \,{\theta }_{i}\end{array}$$

where *r*_*i*_ is a radial distance from a fixed origin, *φ*_*i*_ is a polar angle (or inclination), *θ*_*i*_ is the azimuthal angle, and *i* represents particular set of (*r*, *φ*, *θ*). Since the crystallites were considered to have rotational symmetry along **z** axis, their volume (*V*_*cr*._) was approximated by summing volumes of cylinders stacked perpendicular to **z** as follows:

2 $${V}_{cr\mathrm{.}}=\sum _{i=1}^{n}\pi {(\frac{{x}_{i}+{x}_{i+1}}{2})}^{2}({z}_{i}-{z}_{i+1})$$

wherein *x*_*i*_, *z*_*i*_ represent the Cartesian coordinates of adjacent geometrical elements ordered from the top (*i* = 1) of the crystallite to its maximum diameter (*i* = *n*).

Micromechanical properties were characterized by an instrumented microindentation hardness tester (Micro-Combi Tester; CSM Instruments, Switzerland). For seven individuals, at least 10 indentations were carried out per each cut surface and selected location. In each selected location, the indents were made in the same distance from the EDJ. The indentations were performed with a Vickers indenter; details about experiment geometry have been described elsewhere^34^. The indenter was forced against the cut surfaces with the following parameters: load 1.962 N (200 gf), load time 200 s, and linear loading/unloading rate 0.417 N/s (25,000 mN/min). The high force (200 gf) was chosen in order to obtain large indents (diagonal length >30 *μ*m), which should average possible micro- and nanometer scale inhomogeneities in the enamel structure. The curves showing applied force (*F*) vs. penetration depth (*h*) were used to calculate indentation hardness (*H*_IT_), indentation modulus (*E*_IT_), indentation creep (*C*_IT_), and the elastic part of the indentation work (*η*_IT_) using software Indentation 5.18 (CSM Instruments, Switzerland) according to the theory of Oliver and Pharr^59^.

Fourier transform infrared spectra were recorded using transmission technique (KBr pellets) on a Nicolet 6700 FTIR spectrometer with 2 cm^−1^ resolution and Happ-Genzel apodization in the 400–4000 cm^−1^ region. The width of infrared bands (FWHM) of *ν*_3_ vibrational mode of phosphate anion reflects the combination of the effect of relative particle size of the crystals and the local atomic order. To avoid the effect of particle size, five size-different fractions of three molar types of five selected individuals were measured with respect to Asscher *et al*.^20^ and Poduska *et al*.^60^. The infrared splitting factor (IRSF), a direct indicator of atomic order, or the “crystallinity index,” was then determined using the *ν*_4_ vibrational mode of phosphate anion based on the method according to Weiner *et al*.^61^. The structure of enamel apatite is disordered at the atomic level by the various ions (mainly anions) substitutions. These anion substitutions of enamel apatite lead not only to the variation in physical properties such as stability and solubility but also to the changes of the shape and width of the characteristic *ν*_4_ phosphate vibrational band (originally three times degenerate - *F*_2_ symmetry). The volume of the atomic disorder can be separated from the other significant contribution (particle size, which also significantly influenced the vibrational band character) by the infrared splitting factor (IRSF) plotted^20^ against the full weight in half maximum (FWHM) of the strongest infrared peak (*ν*_4_ – phosphate anion). Crystallinity index of splitting factor (IRSF)^20,60,61^ is mathematically defined as a quotient of a sum of intensity of the two infrared bands originated from the *ν*_4_ phosphate vibration with maxima at 565 and 605 cm^−1^ (*I*_565_ and *I*_605_) divided by intensity of saddle-point (*I*_590_) between these bands, see equation:

3 $$IRSF=\frac{{I}_{565}+{I}_{605}}{{I}_{590}}$$

The maxima of the bands and position of the saddle-point between these maxima were obtained by the bands fitting by Lorentzian function using the spectroscopic OMNIC software^62^. Full weight in half maximum (FWHM) is the parameter of the envelope curve of the split *ν*_3_ (originally three time degenerate vibration; *F*_2_ symmetry) and *ν*_1_ (non-degenerate vibration; *A*_1_ symmetry) phosphate vibrations obtained by fitting in OMNIC software^62^.

The chemical composition (contents of Ca, Na, Mg, P and Cl) of M_1_ and M_3_ was determined using energy-dispersive X-ray spectroscopy. The polished samples were carbon sputtered and the spectra were collected in high vacuum at 20 kV and at working distance of 8–10 mm with an energy-dispersive spectrometer Bruker Quantax 200 attached to a scanning electron microscope Tescan Vega3 XM.

For statistical calculations we used the R language, version 3.0.2 (2013-09-25). A non-parametric Kruskal-Wallis test was carried out to evaluate the inequality of selected variables (see Supplementary Table S1). Shapiro-Wilk test was used to assess whether the data of selected variables are normally distributed (i.e., insignificant P-values point to the Gauss distribution). Then a series of tests was performed to assess the significance of examined variables (summarized in Supplementary Table S2).

### Data availability

The datasets generated during and/or analysed during the current study are available from the corresponding author on reasonable request.

## Electronic supplementary material

Supplementary file
