# Supplementary material for: Enamel apatite crystallinity significantly contributes to mammalian dental adaptations
Source: Sci Rep. 2018 Apr 3;8:5544. doi: 10.1038/s41598-018-23826-0 (PMC5882951; doi:10.1038/s41598-018-23826-0)
Supplement: Supplementary file 1 — Supplementary file [file 41598_2018_23826_MOESM1_ESM.pdf]

Supplementary Information

Enamel apatite crystallinity significantly contributes to mammalian dental adaptations

Anna Kallistová[1,2,*], Roman Skála[1,2], Miroslav Šlouf[3], Petr Čejchan[2], Irena Matulková[4], and Ivan Horáček[5,*]

[1]Institute of Geochemistry, Mineralogy and Mineral Resources, Faculty of Science, Charles University, Albertov 6, Prague 2, Czech Republic
[2]Institute of Geology of the CAS, v.v.i., Rozvojová 269, Prague 6, Czech Republic
[3]Institute of Macromolecular Chemistry of the CAS v.v.i., Heyrovského náměstí 2, Prague 6, Czech Republic
[4]Department of Inorganic Chemistry, Faculty of Science, Charles University, Hlavova 2030/8, Prague 2, Czech Republic
[5]Department of Zoology, Faculty of Science, Charles University, Viničná 7, Prague 2, Czech Republic
[*]ivan.horacek@natur.cuni.cz; kallistova.anna@gmail.com

ABSTRACT

Table S1. P-values of Kruskal-Wallis test (K-Wt) and Shapiro-Wilk test (S-Wt) related to:

	**K-Wt**	**S-Wt**		
		M_1	M_2	M_3
crystallite volume	9.97e^{-08}	0.15	0.66	0.85
microstrain	3.24e^{-07}	0.91	0.4	0.01

Table S2. Null hypothesis and their p-values related to: (a) volume of crystallites $V_{cr.}$; (b) microstrain ε; (c) E_{IT}; (d) η_{IT}; (e) microstructure and its dependence on age; micromechanical parameters (f) H_{IT} and (g) E_{IT} and their dependence on age. Note: $\mathbf{n}_{in.}$ refers to the number of tested individuals; for microstructural experiments 7 M_1 individuals and 5 M_3 individuals were excluded from the study because of lack and/or not mature enamel coating. We used only M_3 to evaluate both microstructure and microhardness and their dependence on the age of an individual (i.e., young (16-30 months) and old (96-108 months) tested groups).

			$\mathbf{n}_{in.}$		
**a)**	$\mathbf{M_1 < M_2}$	$\mathbf{M_2 < M_3}$	$\mathbf{M_1}$	$\mathbf{M_2}$	$\mathbf{M_3}$
Paired t-test	$9.6e^{-05}$	$1.34e^{-05}$	13	20	15
Wilcoxon test	$1.22e^{-04}$	$6.1e^{-05}$			
Kolmogorov-Smirnov test	$2.64e^{-04}$	$1.5e^{-04}$			
**b)**	$\mathbf{M_1 > M_2}$	$\mathbf{M_2 > M_3}$	13	20	15
Paired t-test	$2.49e^{-05}$	$8.94e^{-04}$			
Wilcoxon test	$1.22e^{-04}$	$2.11e^{-03}$			
Kolmogorov-Smirnov test	$8.13e^{-05}$	$2.14e^{-04}$			
**c)**	$\mathbf{M_1 < M_2}$	$\mathbf{M_2 < M_3}$	6*	7*	7*
Paired t-test	$3.05e^{-05}$	$1.03e^{-08}$			
Wilcoxon test	$3.76e^{-05}$	$3.09e^{-07}$			
Kolmogorov-Smirnov test	$3.75e^{-04}$	$1.02e^{-04}$			
**d)**	$\mathbf{M_1 > M_2}$	$\mathbf{M_2 > M_3}$	6*	7*	7*
Paired t-test	0.75	$3.15e^{-10}$			
Wilcoxon test	0.83	$2.4e^{-09}$			
Kolmogorov-Smirnov test	0.58	$1.5e^{-08}$			

**e)**	$\mathbf{V_{cr.}}$	ε		
	**young ≠ old**	**young ≠ old**	**young**	**old**
Paired t-test	0.69	0.7	13	2
Wilcoxon test	0.8	0.85		
Kolmogorov-Smirnov test	0.85	0.81		
**f)**	**young ≠ old**	**young < old**	5*	2*
Paired t-test	$3.24e^{-10}$	$1.62e^{-10}$		
Wilcoxon test	$5.06e^{-10}$	$2.53e^{-10}$		
Kolmogorov-Smirnov test	$1.34e^{-10}$	$3.18e^{-09}$		
**g)**	**young ≠ old**	**young < old**	5*	2*
Paired t-test	$2.2e^{-16}$	$2.2e^{-16}$		
Wilcoxon test	$5.74e^{-12}$	$2.86e^{-12}$		
Kolmogorov-Smirnov test	$8.72e^{-12}$	$8.11e^{-10}$		

* Note: For each individual, at least 10 indentation points were carried out (i.e., at least 60(M_1)/70($M_{2,3}$ and 50(young)/20(old) experimental data were used for statistical calculations).
